# Unpacking the importance of intangible skills in new product development and sustainable business performance; strategies for marketing managers

**DOI:** 10.1371/journal.pone.0238743

**Published:** 2020-09-25

**Authors:** Salman Ali, Guihua Li, Ping Yang, Kramat Hussain, Yousaf Latif

**Affiliations:** 1 Business School & Binhai College of Nankai University, PR, China; 2 School of Economics, Nankai University, PR, China; 3 College of Management and Economics, Tianjin University, Tianjin, P. R. China; DePaul University, UNITED STATES

## Abstract

Firms need sufficient resources (tangible and intangible) and capabilities to build unique products due to customers’ demands and choices, market competition and globalization. Despite sufficient resources, many firms cannot build new products according to the customers’ preferences and market trends due to lack of marketing capabilities, lack of skilled marketing staff and lack of experienced managers. However, studies have not yet examined what types of intangible skills of marketing managers are prominent for building new products. This study examines the importance of the intangible skills; intellectual captial, financial literacy and business experience in new product development that results in sustainable competitive performance. We used a mixed-method approach; questionnaire (283) and interviews (16) for data collection and then applied structural equation modelling for testing the hypotheses. The results revealed that all the three intangible skills; intellectual captial, financial literacy and business experience significant influence new product development and sustainable competitive performance. However, considering the relative importance, financial literacy and intellectual capital are the most significant predictors of sustainable competitive performance and new product development respectively. Moreover, new product development fully mediates the path between intellectual capital and sustainable competitive performance while it partially mediates the link between financial literacy, business experience and sustainable competitive performance. Considering the importance of tangible resources, our study scrutinized that financial resources have a significant influence on new product development and sustainable competitive performance while technological resources do not play a significant role. This research recommends firms to emphasize on the improvement of intangible skills of the managers in order to build new products that result in sustainable competitive position. This study also recommends marketing managers to improve their financial skills and experience by participating in various seminars and workshops that can spur their new idea generation and new product development capabilities.

## 1. Introduction

“If I were down to the last dollar of my marketing budget, I would spend it on PR” Bill Gate

New product development is the key to firm's success, yet the failure rate of new products is very high due to lack of marketing skills and creativity. There is extensive theoretical and managerial concentration on how to lessen the high failure rate of new products. Out of several reasons, the ability of individuals (marketing managers) is deemed substantial [1]. Globalization has brought a threatening competition among organizations either business or non-business, towards the new product development. To build the new products that result in sustainable competitive performance, some managers rely on tangible resources such as adopting a new technology [2, 3] accessing sufficient financial capital [3, 4]), and infrastructure [5, 6], etc. However, others have faith in intangible resources such as intellectual capital [7, 8], networking [9, 10], market knowledge [11, 12], and reputation [13, 14]), etc. Indeed, both the strategies (either building new products via tangible or intangible approach) is useful. For instance, one zone of research has given weight to the tangible factors [2, 3, 6], while others claimed that intangible resources and capabilities are more useful in response to the globalization [15, 16]. Resource-Based View (RBV) also states that a firm needs both tangible and intangible resources for its sustainable competitive position and superior performance [17]. Despite the bunch of studies and evidence, consequences are still fragmented. However, recent studies have given more worth to intangible resources in SMEs sector for several reasons. For instance, SMEs have lack of financial resources and unable to invest in physical means such as new technology [18], extraordinary infrastructure [19] and new product development [20] etc. Moreover, SMEs have lack of support from public, and financial institutions that can hamper their focus in tangible means [15, 16]. As pointed out by Knowledge Based View (KBV) theory, managers with specialized knowledge, intangible skills and intangible capabilities can give maximum benefits and sustainable advantage to organizations [21]. The theory gives more emphasize to knowledge, experience and capability of top management team that are very useful for organization success. Despite a plethora of research, studies are lacking on how marketing managers’ intangible skills help them in developing new products that can spur sustainable position of a firm. Therefore, the present study tests the importance of the intangible skills; Intellectual Capital (IC), Financial Literacy (FL) and Business Experience (BE) in New Product Development (NPD) and Sustainable Competitive Performance (SCP).

Top managers of SMEs often prefer to access external resources via intangible resources, capabilities and skills [15]. Therefore, they indent to hire skilled, capaible and intellectual managers for operational activities such as marketing, finance, export/imports and research [22]. Deliberately, the implications of this research are intended to benefit marketing managers. But alternatively, it facilitates top managers and owners in terms of selecting marketing managers due to their authority role in their organizations. For instance, specifically in SMEs sector, superior authorities are top managers/owners followed by financial, marketing and production managers (unless they are owners).

Considering the importance of intangible resources, recently many scholars especially from emerging economies have shown their research’ interest in intangible factors [23–25]. For instance, Khan, Yang and Waheed [16] scrutinized that intangible resources are considered very prominent for sustainable competitive advantage and superior performance. Favoring the notion, Ying, Hassan and Ahmad [15] claimed that intangible abilities are very crucial for acquiring tangible and intangible resources in a dynamic market. Zhang et al., [26] argued that IC helps spurs new product development performance of business firms either directly or indirectly. IC leads to firms to adopt innovation to gain a stable position in the market [27]. In addition to IC, financial knowledge is very crucial to invest in new ideas. For instance, Tajeddini [28] exposed a significant relationship between financial orientation and new product development in SMEs. Low levels of financial literacy create barriers for SMEs in accessing loan and proper decision making. A study of Study indicates that more than 46.3% managers are unable to access financial services in developing and developed economies due to lack of financial awareness [29]. Alternative, the poor financial literacy may have negatively influence newness of organizations. Similarly, business experience also facilitates managers to make products that are more suitable for the customers [30]. For instance, Mannor, Shamsie and Conlon [31] revealed that experienced managers are better in recognition opportunities and doing new as compared to new and unexperienced managers.

There are several reasons to test the particular intangible skills in NPD and SCP. The first reason of testing the model is the gap, as none of the studies has examined the role of the skills in NPD. Since sufficient tangible resources such as finance, technology, and infrastructure significantly help firms in building new products are an established phenomenon. However, due to lack of evidence, unleashing the importance of intangible resources become the research need. Second, considering the fragmented results of the prior studies, it is worthy to unleash the importance of the intangible skills in NPD. Third, marketing managers are considered one of the most influential resources in any organization [32, 33]. Their strategies, activities, and policies affect product development and sales growth [34, 35]. They are also responsible for sustaining sale growth and gaining a sustainable position in the dynamic market [36, 37]. Since the idea for new products does not come directly but requires marketing skills [38]. Hence, if marketing managers have sufficient skills and knowledge, they will able to recognize new ideas for new products development. However, it is not yet recognized what types of skills are more useful for building new products. For developing new products, marketing managers spend lots of resources to build new products that result high sales growth [39]. However, this study assists them to focus on their intangible skills in the context of NPD that are more meaningful. Fourth, since SMEs face shortage of resources that hinder their innovativeness and survival [18, 40]. This study facilitates them to improve their managers’ skills and knowledge rather then giving more focus to expensive and inconvenience resources. It helps owners and responsible managers of SMEs to improve knowledge and creativity of the marketing managers, so they will be able to build new products that can configure sales growth and sustainable position. Hence, the main objectives of this study are to unbridle the importance of marketing managers intangible skills in building new products that will be launched for securing sustainable competitive position in the markets. Similarly, this study aims to avoid emerging SMEs (e.g. especially Pakistani) from declining sale growth through introducing the skills that may help them in generating new ideas.

This study contributes to the RBV [17] and KBV [21] theories in the context of empirical evidence gathered from marketing managers of SMEs. For instance, the RBV theory enlightens the prevalence of tangible and intangible resources in superior performance [17]. This study emphasizes on intangible skills of marketing managers that are helpful for developing new products. KBV theory sheds light on managers’ knowledge that help them in bringing innovation and monopoly in the markets [21]. In this study, knowledge and experience are considered crucial for NPD that in turn can help firms in securing a sustainable competitive position in the market.

## 2. Theory and literature review

### 2.1 Intellectual capital and sustainable competitive performance

According to the KBV theory [21], managers intangible skills, knowledge and capabilities integrate and assimilate resources in an effective way that give sustainable advantage to a firm. Hence, business firms have now emphasized on IC because it does not only spur financial performance but also significantly facilitates non-financial performance, innovative performance and sustainable competitive advantage [41–43]. Despite the mentioned advantages that can be achieved through one or three dimensions of IC; human capital, relational capital and structural capital, organizations also improve their environmental and economic performance through IC [44, 45]. Particularly in small firms as they have very scared resources, IC has been considered one of the most beneficial resources to maximize the profitability and gain superior performance [16, 42]. In the current knowledge base approaches, human capital, organization and structural capital are considered prominent intangible assets for supply chain integration and competitive advantage [46]. IC is not only important for manufacturing and trading firms. Services firms can also get useful advantages of IC. For instance, Li and Liu [47] claimed that IC helps services organizations in identification of problems that in turn can give sustainable competitive advantage and superior performance. Similarly, Sardo, Serrasqueiro and Alves [48] also claimed that SMEs hotels improve their financial performance through IC. Studies have confirmed that all the dimensions of IC significantly configure sustainable competitive performance of business firms ([46, 49]. Therefore,

*H1*. *Marketing Managers with high IC will significantly contribute to sustainable competitive performance*

### 2.2 Financial literacy and sustainable competitive performance

Financial literacy is very important particularly in small firms because the decision-making process of small firms is directly influenced owners and managers [50]. It significantly affects overall performance of creative ventures [51]. In SMEs sector, the role of financial literate managers is prominent for understanding customers’ needs and their necessities. Hence these advantages provides high profits [52]. Financial literate person understand important financial terms and concepts. They confidently handle their financial resources in an effective way to make useful financial decision and do long term financial planning [53]. Financial knowledge, financial skills and financial information are very crucial for managers (financial and marketing) in SMEs because they facilitate in acquiring external finance that is the key to competitive advantage [54]. In emerging economies, SMEs sector get sufficient benefits of financial literate managers as they can effectively manage resources for high performance [55]. For instance, Xu, Shi, Rong and Yuan [56] claimed that financial literate managers can easily access credit in emerging economy China. Hence, it can be argued that marketing managers with financial education will easily acquire external knowledge related to market, customers and products that in turn will spur business performance. Managers with lack of financial knowledge, lack of financial management practices and lack of financial behaviors make wrong decisions that can damage their business operation [57]. All these insufficiencies are very harmful for business firms and their reputations in the markets. Hence, it today business era, financial skills is very essential for newness and effectiveness [58]. Hence,

*H2*. *Marketing Managers with high FL will significantly contribute to sustainable competitive performance*

### 2.3 Business experience and sustainable competitive performance

Managers who have worked in international organizations and have gained foreign exposure and experience have superior ability over other managers in term of export performance and innovation [59]. Firms should consider experienced managers in decision making process because ignoring experience may lead to competitive disadvantage [60]. Experienced managers can easily secure sustainable position in the market and can improve firm performance because they have the ability to manage the resources and a better way as compared to the firms where managers have lack of experience [61]. Individuals with entrepreneurial knowledge and experience have better understanding of running business. They know the acquisition of knowledge and management of resources that are essential for success of newly established entrepreneurial ventures [62]. Managers with prior experience in different organizations give benefits to the present firms in several ways. For instance, they acquire and assimilate knowledge in a better way to lead the firms towards competitive advantage [63]. Consider the benefits of marketing experiences, for instance, managers who have broad experience in marketing drive their sale growth rapidly and spur their firm’s profitability [60]. Anderson, Chandy and Zia [64] tested the relative importance of marketing and financial skills in business performance and profitability. They revealed that both types of skills are very essential for high performance but compared to financial skills, marketing skills provide greater advantages in term growth and profitability for entrepreneurial firms. Therefore;

*H3*. *Marketing Managers with high BE will significantly contribute to sustainable competitive performance*

### 2.4 Intellectual capital and new product development

Intellectual capital is the composition of knowledge, skills and ideas that are used by managers for organizational activities. It benefits organizations in several ways such as new product innovative performance ([26, 41], competitive advantage [42, 49] and economic performance [44, 45] etc. IC is the valuable source of product innovation and managerial innovation business sectors [38]. For instance, Eiteneyer, Bendig and Brettel [65] claimed that organizations used social capital (a dimension of IC) to improve their new product innovative performance in the turbulent markets.

NPD is a new activity that may need skills, capabilities and competencies. Hence, it is probably right that intellectual managers will help firms in building new products. For instance, Molodchik and Jardon [66] claimed that IC is a significant factor of product novelty in SMEs sector and can significantly increase the probability of transition to a new market level. There are several studies that have tested the relationship between IC and innovation. Subramaniam and Youndt [67] tested the influence on human, organizational and social capital on innovation and revealed a positive relationship. However, the study of Delgado-Verde et al. [68] shown a significant relationship between human capital and innovation while it show an insignificant association between structural and organization capital and innovation. Hence,

*H4*. *Marketing Managers with high IC will significantly contribute to new product development*

### 2.5 Financial literacy and new product development

Financial literacy is the capability of a business to properly oversee financial resources over the life cycle and strengthen the link with financial services and financial products. In strategic decision-making process product modification, business managers need to be rationale and have reasonable degree of knowledge and information (financial literacy) in order to develop valuable products. However, it is unfortunate that all the SMEs have not financially literate managers. For instance, Ayyagari, Beck and Demirguc-Kunt [69] claimed in their study that if SMEs managers are not familiar with financial terms or not able to build innovative products, they will not need to retain them. It argues that financial literacy is every essential for right decision making and new product development. For instance, several studies have claimed that individuals and SMEs managers with a low level of financial literacy often dislike to participate in formal financial system, borrow at a higher interest rate and make wrong decision relative to their more financially literature peers (Lusardi, Mitchell and Curto [70] and Lusardi and Tufano [71]. Managers with lack of financial skills, knowledge and concepts are unable to build new products. In other words, lack of financial literacy hamper managers from building new products and new services. Hence, business firms must emphasize on financial skills of their marketing managers to build new products and services [58]. Therefore;

*H5*. *Marketing Managers with high FL will significantly contribute to new product development*

### 2.6 Business experience and new product development

Managers with experience are easily introduce innovative activities and contribute to innovative performance of firm. In contrast, lack of experienced managers fail to adopt innovative process for operational activities [72]. In a turbulent and competitive environment, firms need experienced managers to enhance their innovative activities and innovative performance [73]. In general, when SMEs aim to build new product and adopt innovation, they get benefits of the managerial skills such as education, intellectuality and experience [74].

In emerging economies such as China, NPD is a challenging task for marketing managers. In response to this, marketing knowledge and experience is the critical strategic solution to produce a unique product for the market [75]. Decision making about new product development requires marketing skills and competencies. Hence, marketing managers should be competent in marketing information and experience [76]. Suwannaporn and Speece [77] also pointed that that marketing knowledge, experience and information are the most important factors in the success of new product development. Market knowledge and marketing capabilities are required for any marketing related task such as R&D and innovation [78]. Experience helps manufacturing firms in making efficient decision-making and building new and innovative products for customers and markets [79]. For instance, Roy and Islam [80] claimed that firms with advanced technology and experienced managers build more innovative products for customers as compared to those firms which have lack of modern technology and lack of experienced marketing managers. Hence, it posits that business experience of marketing managers enables them in building new and unique products for new and existing customers. Hence, we posit that,

*H6*. *Marketing Managers with high BE will significantly contribute to new product development*

### 2.7 New product development and sustainable competitive performance

Customers always desire to purchase new products that have innovative features. Hence, it is the responsibility of the marketing managers to offer such products that can satisfy need and wants of the customers [81]. As compared to developed market firms, emerging firms have lack of competency in response to dynamic market conditions. Hence, managers innovative product capability significantly helps emerging firms to sustain their performance in the turbulent market [82]. Companies manage their resources for product innovation process because it in turn improves performance [83]. Innovative product is the key of success in business firms [1]. As a firm announces new products and new brands, customers get signals and intend to purchase that improves the firm values [84]. New product development does not only improve financial performance of firms but also plays a significant role in the success of economic and supply chain performance [85]. Different types of innovative products and innovation play worthy role in firm performance [86]. Therefore, we posit that,

*H7*. *New product development significantly contributes to sustainable competitive performance*.

### 2.8 Mediating role of new product development

SCP is the major goal of every business firm. To be succeeded in the goal, a firm needs to consider several factors such as environment, markets, and innovation. One of the best strategies of business firms is to improve their IC that may help in innovation and performance [87]. It posits that IC does not directly improve competitive performance, but it first configures the internal processes and strategies of a firm that, in turn enhance performance. For instance, several studies have favored the indirect relationship between IC and performance via competitive advantage [16, 42], innovation [88], and resource acquisition [15], etc. Intellectual managers have broad knowledge of markets, customers and technology; hence they transform the knowledge into innovative ideas (building new products) that positively contribute to firm performance [89]. In SMEs sector, the role of the intangible factor (IC) is not underestimated. It is considered one of the most significant elements of innovative performance and innovative products that can spur competitive performance [41]. Firms use their IC to build and transform their innovative work behaviors into performance [90].

Owners and managers of small firms will able to identify and recognize new opportunities if they have sufficient financial knowledge. In contrast, lack of financially literate managers often miss to recognize newness that in turn negatively affects their business performance [91]. Organizations that have robust financial management practices spur competitive advantage in SMEs that in turn enhance financial performance [92]. Financial literate managers are the central players of SMEs who configure the internal process and operational activities (e.g. related to product development) in a way to gain maximum benefits [93]. Financial literature managers reduce a variety of cost in different way and produce unique kinds of products for customers that in turn enhance their sale growth and profitability [94].

Experience managers accumulate knowledge related to market, products and customers which help them in decision making process and significant influence business performance [95]. For competitiveness and survival, business organizations need innovative products. However, it is necessary and essential for business firms to focus on experience managers who can easily developed innovative products and bring innovative ideas [96]. Candi, Beltagui and Riedel [97] in their study strongly recommended experienced managers for SMEs due to their significant role in new product development and innovation that are vital for competitive advantage. Firms’ performance is significantly influence by managers experience [98]. Managers who have worked in different business cultures are more opportunistic and enjoy high performance [99]. Managerial experience differentiates firms from one another in term of sustainable competitive advantage and performance. Because experienced managers are able to acquire useful resources and have more knowledge of external markets to seize opportunities ([95]. Learning from experience facilitates their capacity to manage resources in a useful way that can give high profit and sustainable competitive advantage [100]. As stated earlier that experienced managers are aware of trade rules, market trends and market growth. Hence, recognition of new profitable opportunities and new idea generation is easy for them [101]. Business experience helps managers in strategic planning, learning orientation, recourse management, communication and entrepreneurial activities that in turn enhance performance [102, 103]. Therefore, we proposed the following hypotheses;

*H8*. *New product development mediates the path between IC and sustainable competitive performance*

*H9*. *New product development mediates the path between FL and sustainable competitive performance*

*H10*. *New product development mediates the path between BE and sustainable competitive performance*

**Fig 1:**
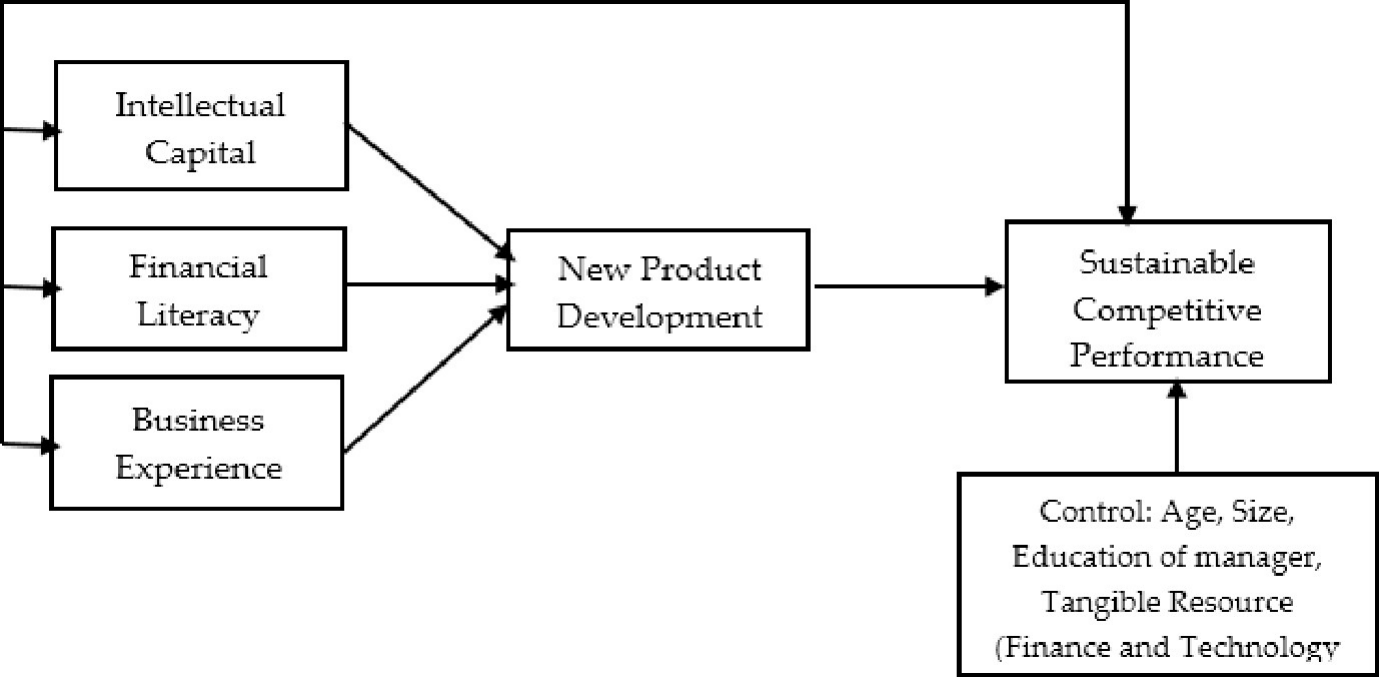
Research model.

Fig 1. Illustrates the research model.

## 3. Methodology

### 3.1 Research design, sample and data

The aim of this study was to unleash the importance of marketing managers’ intangible skills; IC, FL, and BE in NPD and SCP in Pakistani SMEs. One of the major reason behind choosing Pakistan is the geographical benefit as the country is located in the best trade location “Asian and Europe” [104]. Moreover, the country has strong trade ties with Asian (Japan, China, India and Afghanistan etc) and Europe (UK, USA, Australia and Germany etc.). Considering the geographical benefits and trade connections, it is argued that Pakistan has many similarities of marketing strategies and business activities with other nations [105]. Hence, implications based on Pakistani evidence might be suitable for other countries and markets. Moreover, despite having a significant role of marketing managers in product development and business success, studies have not yet considered the role of the specified intangible skills. Motivated by these opportunities, we realized that evidence from Pakistan industry will provide worthy implications.

The nature of the research is quantitative, and the deductive approach is used to test the hypotheses. The unit of analysis for this study was marketing managers of Pakistani SMEs. Because in Pakistani firms, marketing department is responsible to build and develop new products for customers. Data were gathered through a survey method where a structured questionnaire was used. The questionnaire was prepared in English language because the language is easily understood by Pakistani managers. Moreover, the official language in the busuiness industry is English. The questionnaire is adopted from previous studies (see S1 Appendix) and Nankia university research committee approved it for data collection. The participants were asked that the survey is volunteer and you are not compelled to fill it compulsory. However, due to collectivism culture in Paksitan, most of the businesses are likely help and cooperative in data collection. Hence, the participants consent was waived by the committee as and no permsission of verbal or written statement was required. However, to reduce the biases, we have mentioned in the cover letter of the questionnaire that the data of this study are used only for research purpose and the firm ‘information will not be published elsewhere. We obtained the registered SMEs lists chamber of commerce and industry located Islamabad, Rawalpindi and Lahore. The reasons of choosing these cities are; (a) these are the biggest business cities, (b) head offices of the SMEs are located in these regions and (c) formal activities regarding product development, internationalization and strategy are carried out in these regions [22, 104]. We randomly selected 283 SMEs as a sample for data collection and contacted through a hard copy questionnaire. We distributed total 600 questionnaires of which 200 in each city. As per the study requirements, we requested marketing department of every firm to participate in the survey. Due to the busy schedule of the managers, many firms did not return questionnaire on the time (e.g. after distribution). Hence, we followed back the questionnaires after one to two weeks. During two months, we received 111 useable questionnaires from Islamabad industrial sector, 90 usable responses from Rawalpindi industrial sector and 82 useable questionnaires from Lahore with response rates 39.22%, 31.80% and 28.98% respectively. Hence, the desired sample of 283 gained within two months with an effective response rate 47.17%.

The description and demographic detail of the marketing managers and firms have shown in Table 1. It indicates that there were 60 firms where 20 to 50 employees were working, 56 firms were those where 51 to 100 employees were engaged in operational activities. There were also 56 firms where 101 to 150 labors were hired. In the sample, 51 firms have 151 to 200 employees while 60 firms have those where 201 to 250 employees were busy in operational activities. There were 108 managers who were 20 to 35 years old, 95 managers were 36 to 45 years old while 80 managers were 46 to 60 years old. We also asked the educational background of the marketing managers in the survey and revealed that majority of the marketing managers have bachelor (100 managers) and master (88 managers) degrees while 46 were intermediate and 42 were MS. Only 7 managers were PhD in this study.

**Table 1:** Managers and firms information.

Description	Frequency	Percentage
Size of firms		
1. 20–50 employees	60	21.2
2. 51–100 employees	56	19.8
3. 101–150 employees	56	19.8
4. 151–200 employees	51	18.0
5. 201 to 250 employees	60	21.2
Age of Managers		
1. 20 to 35 years	108	38.2
2. 36–45 years	95	33.6
3. 46 to 60 years	80	28.3
Educational Background		
1. intermediate and less	46	16.3
2. Bachelor	100	35.3
3. Master	88	31.1
4. MS / MPhil	42	14.8
5. PhD	7	2.5
Total	283	100

### 3.2 Measurement of the constructs

Intellectual Capital: in some studies, the intangible asset was used for IC [106, 107]. In general, studies have discussed three major dimensions of IC named human capital, structural capital and relational capital [16, 42]. In this study, we used 6 items e.g. adopted from [15, 42] in which all the three dimensions are covered. A representative item shows “Benchmarking strategy knowledge against that of competitors.”

Financial Literacy: in the literature, similar words such as financial knowledge, financial education, and business literacy are used for financial literacy. The major aim of financial literacy is how a manager knows the financial terms and indicators that are useful for his business. In some business studies especially from investment perspective, financial literacy is measured by asking interest rate, inflation and future income [108, 109]. However, in case of SMEs, studies have recommended the questions related to financial management, future savings, and preparing financial statement, etc. for the firm [15, 94]. Hence, in this study, we also relied on the managers’ financial literacy, and the measures (13 items) were adopted from Ying, Hassan and Ahmad [15] where the items are validated. A sample item displays “We can prepare basic books of accounts.”

Business Experience: It demonstrates the experience of managers in the purchase, sales, import and export and adverting, etc. The measures of business experience are adopted from Ying, Hassan and Ahmad [15], who have used 5 items in the context of SMEs. A sample item displays “I have good experience in export and import business.”

New Product Development: a firm has several strategies for NPD. For instance, building a new product, modification in an existing product and adding a new line to an existing product line, etc. In this study, we used seven comprehensive measures of NPD that are adopted from the prior study of Cui and Xiao [110]. A sample question that has managers is “In the recent years, we have developed a totally new product to the world that opened up a brand-new market.”

Sustainable Competitive Performance: measurement of performance is easy in the case of listed firms as financial data of the firms can be easily accessed. However, in case of SMEs, it is difficult to obtain financial information and financial data. Hence, researchers believe in self-reported measures for performance when they research SMEs [42, 111]. We also used self-reported approach to measure SCP of the SMEs adopted from [15, 18] where managers were asked how your firm performs since the last 3 years in term of return on equity, return on investment and return on assets, etc. as compared to the industry rivals and major competitors. The scales were used strongly declined 1 to strongly improved. However, for other variables such as IC, FL, BE, and NPD, we used five points Likert scales stating strongly disagree 1 to strongly agree 5.

### 3.3 Control variables

Control variables help researchers in reducing the spurious results. Hence, we controlled the age of the managers, size of the firms, educational background and tangible resources available to the firms. We scrutinized mixed results for the control variable in the structural models. All the results have discussed in detail with each model.

## 4 Data analysis and results

### 4.1 Descriptive statistics

We tested normality, multicollinearity, means, and Standard Deviation (SD) of the data set that are shown in Table 2. We revealed that our data are normally distributed as all the factors have their skewness and kurtosis values below the cutoff ±2 as recommended by Byrne and Van de and Vijver [112]. Our data also have no threat of multicollinearity as the Variance Inflation Factor (VIF), and tolerance values are in the acceptable range (below 3 and above 0.10 respectively) [113]. Business experience has the highest means value 3.83 while IC has the lowest means value 2.17. NPD has the highest SD 0.47, while IC has the lowest SD = 0.27.

**Table 2:** Descriptive statistics.

Variables	Mean	S.D.	Skewness	Kurtosis	SCP		NPD	
					Tolerance	VIF	Tolerance	VIF
Intellectual Capital	2.7128	0.27430	-0.506	1.915	0.785	1.274	0.822	1.216
Financial Literacy	3.3092	0.38002	-0.016	1.843	0.575	1.740	0.598	1.673
Business Experience	3.8350	0.43224	-0.136	0.344	0.569	1.758	0.611	1.637
Product Development	3.6161	0.47161	-0.227	0.629	0.683	1.465	-	-
Competitive Performance	3.5188	0.38837	-0.180	1.892	-	-	-	-

### 4.2 Common method bias

Scholars have criticized cross-sectional data due to the common method bias [114]. Hence, to check if our data are threatened by the problem, we applied Harman one factor test (using exploratory factor analysis and principle component factor) in SPSS. Our results revealed 5 factors that explained the total variance of 69.808% of which the first factor displayed only 38.181% variance which is less than 50%. Hence, we revealed that the common method bias does not influence our results. In addition to this method, we also executed the influence of common latent factor on the measurement model. We compared the results of the both models (one with common latent factor and other is measurement model) and scrutinized the absence of the threat.

### 4.3 Correlations

Table 3 shows the correlation coefficients between the constructs. It illustrates that IC, FL and BE are significantly related to SCP (r = 0.442, p < 0.01, r = 0.686, p < 0.01 & r = 0.620, p < 0.01) and NPD (r = 0.387, p < 0.01, r = 0.466, p < 0.01 & r = 0.490, p < 0.01) respectively. NPD has a significant relationship with SCP (r = 0.546, p < 0.01). Both financial and technological resources are significantly associated with SCP (r = 0.446, p<0.01 & r = 0.339, p < 0.01) and NPD (r = 0.325, p < 0.01 & r = 0.203, p < 0.01) respectively. To summarize, the correlation between all the factors show significant results.

**Table 3:** Correlation coefficients.

Variables	1	2	3	4	5	6	7	8	9	10
1. Size	1									
2. Age	0.117*	1								
3. Education	-0.020	0.197**	1							
4. Financial Resource	0.061	0.230**	0.167**	1						
5. Technology Resources	0.023	0.215**	0.067	0.244**	1					
6. Intellectual Capital	-0.015	0.213**	0.195**	0.231**	0.171**	1				
7. Financial Literacy	0.098	0.314**	0.172**	0.352**	0.391**	0.390**	1			
8. Business Experience	0.036	0.304**	0.163**	0.309**	0.384**	0.364**	0.608**	1		
9. Product Development	0.071	0.205**	0.076	0.325**	0.203**	0.387**	0.466**	0.490**	1	
10.Competitive Performance	0.140*	0.459**	0.273**	0.446**	0.339**	0.442**	0.686**	0.620**	0.546**	1

*. Correlation is significant at the 0.05 level (2-tailed).

**. Correlation is significant at the 0.01 level (2-tailed).

### 4.4 Confirmatory factor analysis

To check if the model is fit and the items and constructs have satisfactory regression weight, validity, and reliability, we executed confirmatory factor analysis. In the first phase, we checked the fitness of the model and ensured that all criteria of the model fit (see Table 4); x/df, RMR, RMSEA, GFI, AGFI and NFI, CFI and TLI are satisfactory as suggested by the scholars [115, 116]. All the regression weights of the items were significant (p < 0.001) toward their respective items. In addition to model fits, we checked the convergent validity (see Table 5) of the factors and revealed that all the constructs have satisfactory value (above 0.50) as per the suggestion of Fornell and Larcker [117]. Discriminant validity of all the factors also gave desirable value (above 0.70) as recommended by Fornell and Larcker [117] Additionally, we tested the composite reliability and confirmed that the items are reliable as all the factors have their composite reliability greater than 0.70 [118].

**Table 4:** Model fits.

Models	X/DF	RMSEA	RMR	GFI	AGFI	NFI	TLI	CFI
Measurement Model	2.384	0.070	0.016	0.81	0.80	0.835	0.89	0.90
Structural Model1	2.470	0.072	0.051	0.83	0.81	0.81	0.86	0.88
Structural Model2	2.312	0.068	0.047	0.80	0.80	0.81	0.87	0.88
Structural Model3	2.996	0.079	0.055	0.86	0.81	0.90	0.92	0.93
Structural Model4	2.305	0.068	0.045	0.80	0.80	0.81	0.87	0.88

**Table 5:** Standardized loading, validity, and reliability.

Items and Variables	Estimate	AVE	√AVE	C.R.
Intellectual Capital				
Item6	0.68***	0.50	0.70	0.85
Item5	0.76***			
Item4	0.70***			
Item3	0.71***			
Item2	0.64***			
Item1	0.72***			
Financial Literacy				
Item13	0.71***	0.51	0.71	0.92
Item12	0.72***			
Item11	0.73***			
Item10	0.78***			
Item9	0.71***			
Item8	0.80***			
Item7	0.60***			
Item6	0.77***			
Item5	0.70***			
Item4	0.58***			
Item3	0.82***			
Item2	0.68***			
Item1	0.61***			
Business Experience				
Item5	0.90***	0.58	0.76	0.87
Item4	0.76***			
Item3	0.73***			
Item2	0.65***			
Item1	0.75***			
New Product Development				
Item7	0.84***	0.73	0.85	0.95
Item6	0.80***			
Item5	0.82***			
Item4	0.85***			
Item3	0.86***			
Item2	0.92***			
Item1	0.89***			
Competitive Performance				
Item8	0.83***	0.72	0.85	0.95
Item7	0.84***			
Item6	0.82***			
Item5	0.90***			
Item4	0.78***			
Item3	0.92***			
Item2	0.85***			
Item1	0.83***			

*** = significant at (0.001), AVE = Average Variance Extracted, CR = Composite Reliabiltiy

### 4.5 Structural model

We executed a structural model to test the hypotheses of the research. For the purpose of obtaining useful insights, we tested several models for the direct paths and indirect paths between the marketing managers’ intangible capabilities and SCP. The results of the direct models are shown in Table 6.

**Table 6:** Hypotheses testing (direct relationship).

Structural Model 1			Estimate	S.E.	C.R.	P
CompPerform	<—	IntCapital	0.150	0.067	2.230	0.026
CompPerform	<—	FinLiteracy	0.334	0.060	5.562	0.000
CompPerform	<—	BusExp	0.186	0.049	3.836	0.000
CompPerform	<—	Age	0.109	0.020	5.333	0.000
CompPerform	<—	Education	0.041	0.016	2.563	0.010
CompPerform	<—	Size	0.021	0.011	1.913	0.056
CompPerform	<—	FinResource	0.085	0.019	4.559	0.000
CompPerform	<—	TechResourc	0.009	0.021	0.407	0.684
Structural Model 2						
ProdDevelp	<—	IntCapital	0.300	0.108	2.773	0.006
ProdDevelp	<—	FinLiteracy	0.248	0.090	2.743	0.006
ProdDevelp	<—	BusExp	0.284	0.077	3.673	0.000
ProdDevelp	<—	Age	0.009	0.032	0.278	0.781
ProdDevelp	<—	Education	-0.029	0.026	-1.135	0.256
ProdDevelp	<—	Size	0.013	0.018	0.726	0.468
ProdDevelp	<—	FinResource	0.095	0.030	3.180	0.001
ProdDevelp	<—	TechResourc	-0.026	0.035	-0.743	0.458
Structural Model 3						
CompPerform	<—	ProductDev	0.283	0.040	7.068	0.000
CompPerform	<—	Size	0.021	0.012	1.810	0.070
CompPerform	<—	Education	0.060	0.017	3.539	0.000
CompPerform	<—	Age	0.136	0.022	6.182	0.000
CompPerform	<—	TechResourc	0.075	0.024	3.170	0.002
CompPerform	<—	FinResource	0.097	0.021	4.752	0.000

#### 4.5.1 Structural model 1

**Fig 2:**
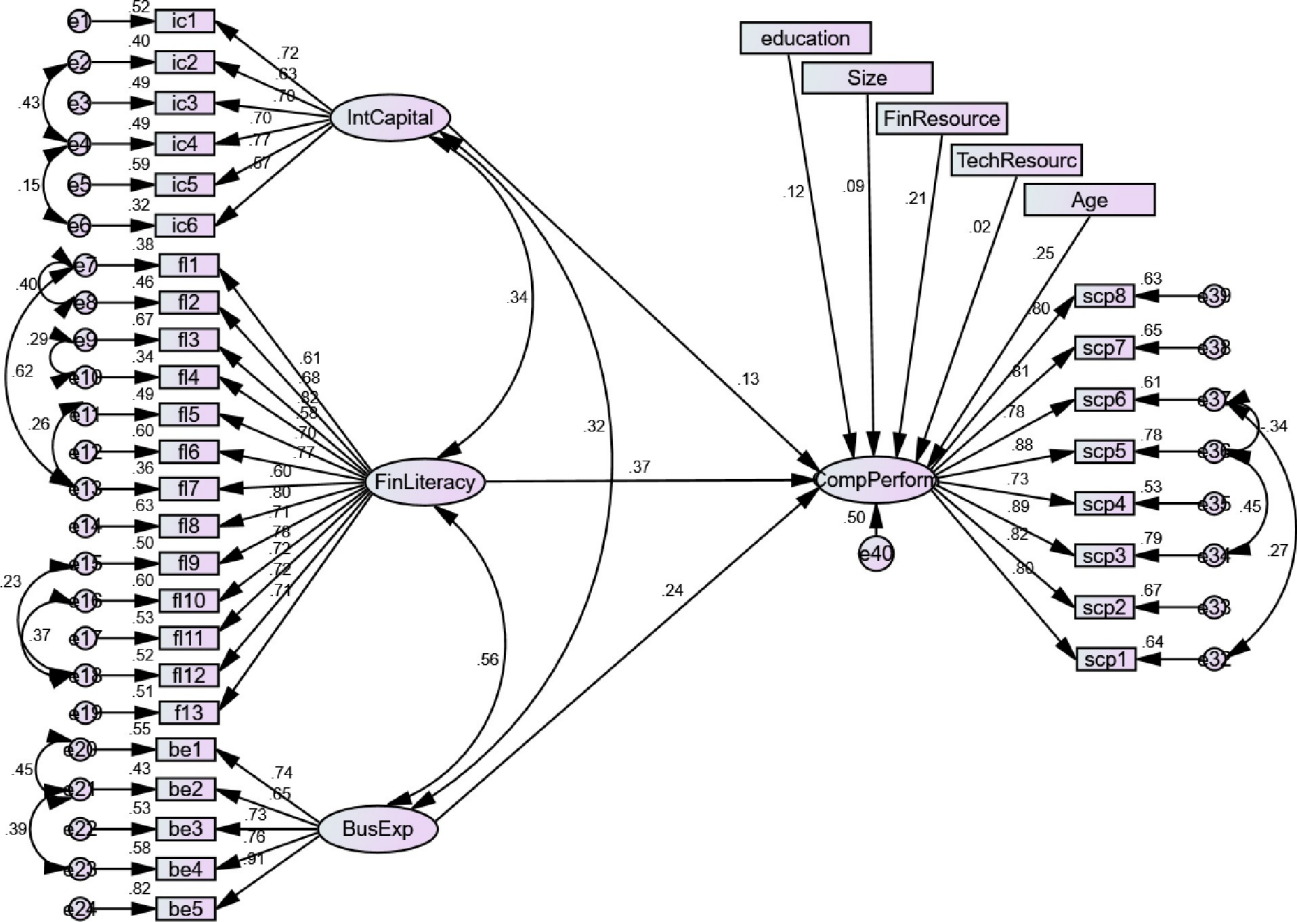
Structural model 1.

The model (see Fig 2) is performed to check the influence of IC, FL and BE on SCP in the presence the control factors, educational background, size of firms, and age of managers, financial, and technological resources. We ensured the fitness of the model in terms of x/df, GFI, AGFI, NFI, TLI, RMSEA, and RMR, as suggested by Kline [116].

Our findings revealed that IC, FL and BE have a significant influence on SCP (β = 0.150, C.R. = 2.230, P = 0.026, β = 0.334, C.R. = 5.562, P = 0.000 & β = 0.186, C.R. = 3.836, P = 0.000) which supported H1, H2 and H3 respectively. In the control variables, the only size of firms and technological resources have an insignificant role while age of managers, educational background and financial resources play a significant role in the model. IC, FL and BE collectively explain 50% variance in SCP when controlled for the factors; educational background, technology, and financial resources, size, and age of the managers.

#### 4.5.2 Structural model 2

**Fig 3:**
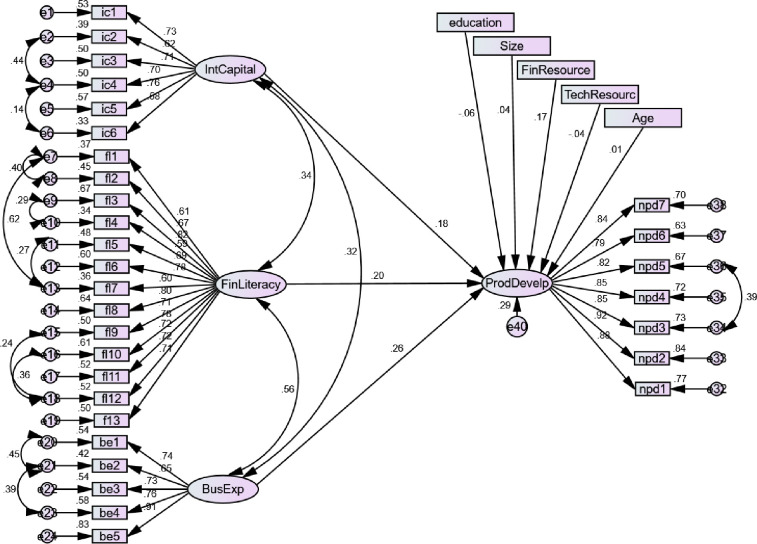
Structural model 2.

In this model (see Fig 3), we executed the effect of IC, FL, and BE on NPD in the availability of the control factors. All the criteria of the model fits (x/df, GFI, AGFI, NFI, TLI, RMSEA, and RMR) were achieved as per the recommendation of Kline [116].

The results describes that IC, FL and BE have a significant influence on NPD (β = 0.300, C.R. = 2.773, P = 0.006, β = 0.248, C.R. = 2.743, P = 0.006 & β = 0.284, C.R. = 3.673, P = 0.000) which supported H4, H5 and H6 respectively. In the control factors, the age of managers and size of the ventures, educational background, and technological resources show insignificant effect while only financial resources display significant influence on NPD. Only 29% variation is explained by IC, FL and BE in NPD when controlled variables are considered.

#### 4.5.3 Structural model 3

**Fig 4:**
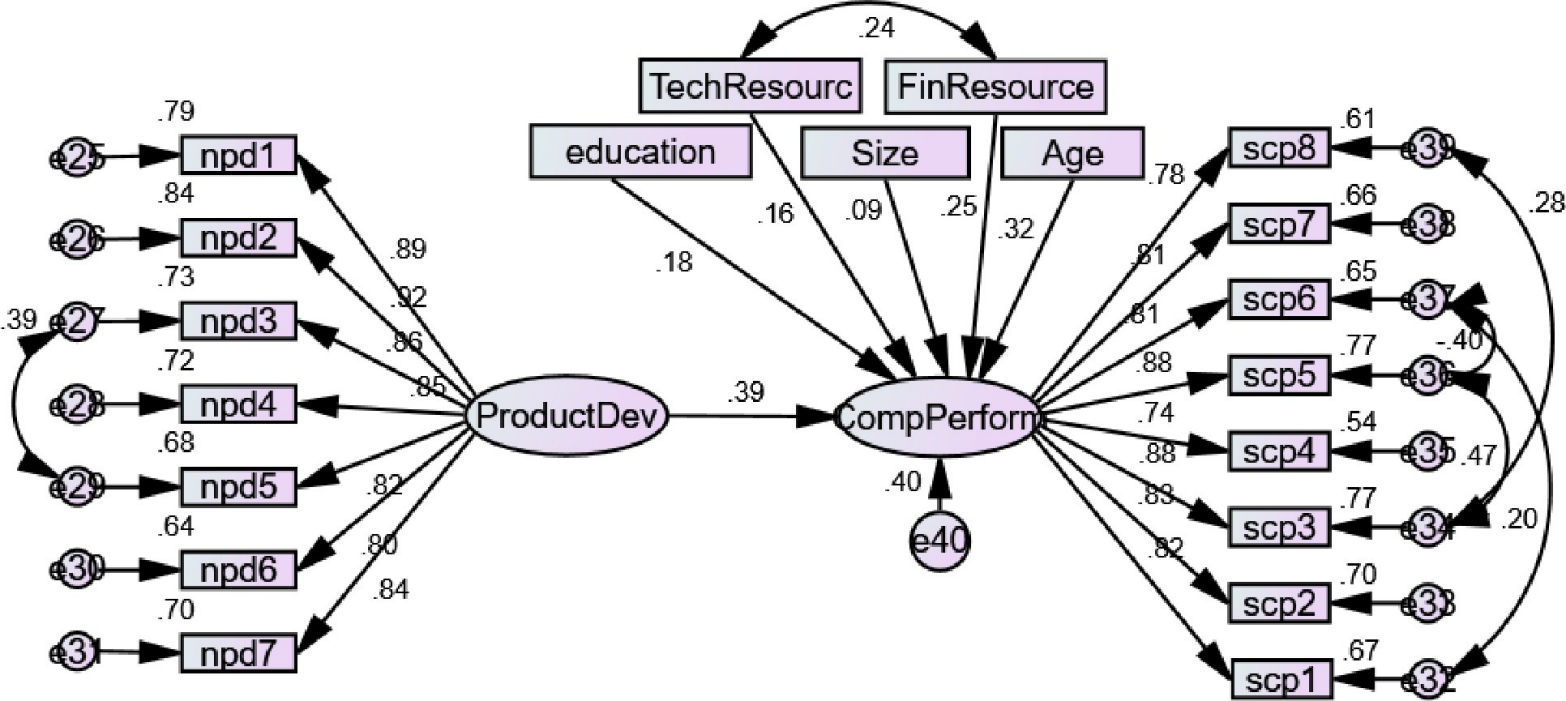
Structural model 3.

This model (see Fig 4) examines the influence of NPD on SCP in the presence of the discussed control factors. The model is satisfactory fits as the values of x/df, GFI, AGFI, NFI, TLI, RMSEA, and RMR met the criteria suggested by Kline [116].

The results displayed that NPD has a significant influence SCP (β = 0.283, C.R. = 7.068, P = 0.0.000), which favored H7. All the control factors show significant role despite size of firms. R square reveals that 40% variance in SCP is explained by NPD in the presence of the controlled factors.

#### 4.5.4 Structural model 4

**Fig 5:**
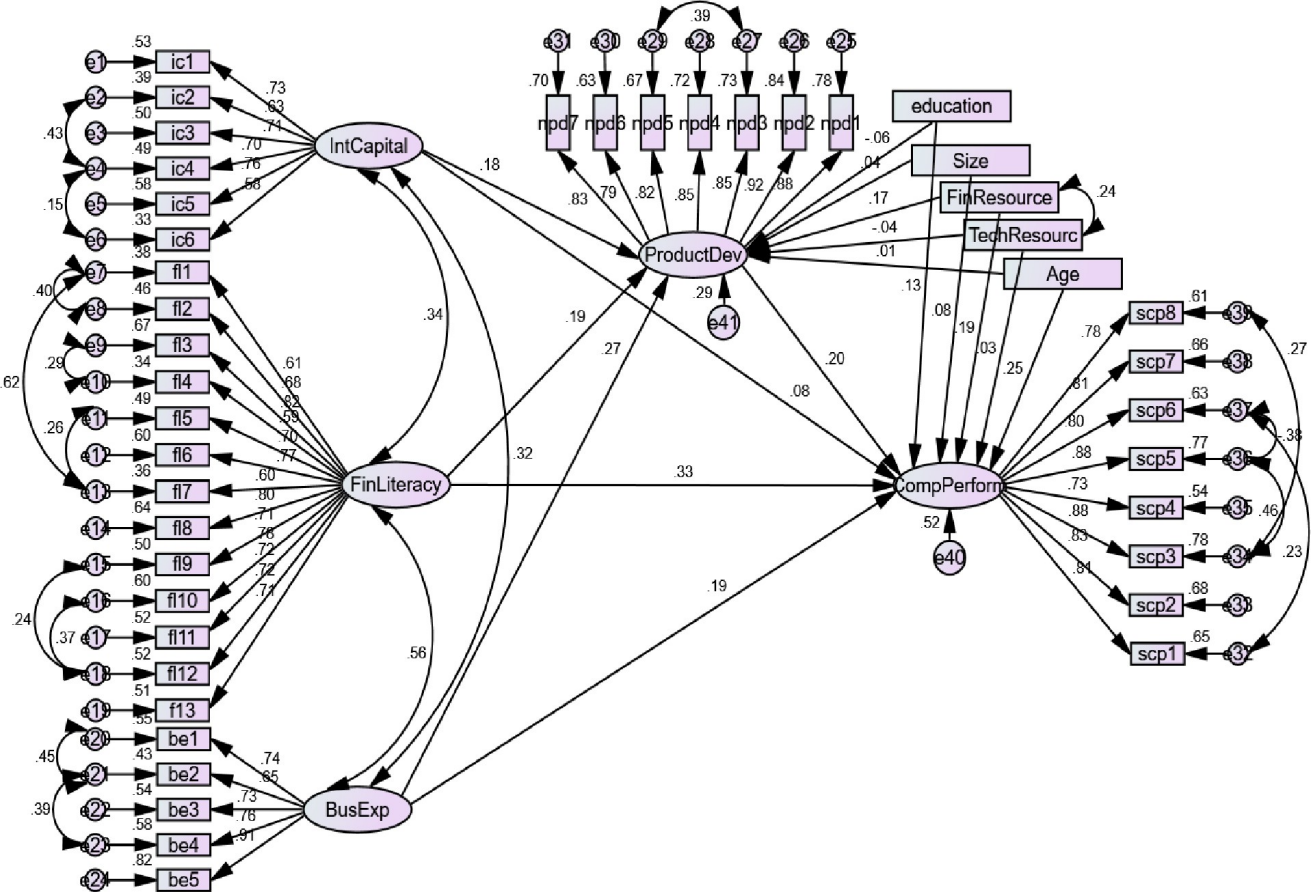
Structural model 4.

This is the main model (see Fig 5) in which the mediating role of NPD between the intangible skills; IC, FL, and BE and SCP has been tested in the presence of the control variables; size of firms, educational background, age of marketing, technological and financial resources. The entire model fits x/df, GFI, AGFI, NFI, TLI, RMSEA, and RMR were in the acceptable range [116].

The results (see Table 7) demonstrated that the indirect influence of IC on SCP via NPD is significant (β = 0.037, p = 0.023) while the direct influence of IC on SCP became insignificant (β = 0.081, p = 0.196) that reveal a full mediating role of NPD. Hence, H8 is fully supported as NPD fully mediates the path between IC and SCP. The indirect influence of FL on SCP through NPD is also significant (β = 0.040, p = 0.022), but the direct influence also remained significant (β = 0.328, p = 0.001), which revealed the partial mediating role of NPD and hereby partially supported H9. Moreover, BE shows indirect significant effect on SCP (β = 0.054, p = 0.012) but the direct influence also remained significant (β = 0.185, p = 0.025) which confirmed the partial mediation of NPD and thus partially supported H10. R square of the structural model 4 indicates 52% variance in SCP and 29% variance in NPD that is explained by the intangible skills; IC, FL, and BE in the existence of the control factors. In the control factors, only size of firms and technological resources have insignificant role in SCP, while only financial resources have a significant role in NPD.

**Table 7:** Hypotheses testing (indirect relationship).

hypotheses	Direct effects	Indirect effects	Total effects
CompPerform← Intellectual Capital (via NPD)	0.081(0.194)	0.037(0.023)	0.118(0.069)
CompPerform←Financial Literacy (via NPD)	0.328(0.001)	0.040(0.022)	0.368(0.001)
CompPerform←Business Experience (via NPD)	0.185(0.025)	0.054(0.012)	0.239(0.002)
Product Devep← Intellectual Capital	0.182(0.029)	-	-
Product Devep← Financial Literacy	0.195(0.026)	-	-
Product Devep← Business Experience	0.266(0.010)	-	-
CompPerform←Product Development	0.204(0.013)	-	-
CompPerform← Size of firms	0.083(0.074)	-	-
CompPerform←Age of managers	0.248(0.001)	-	-
CompPerform←Educational Background	0.130(0.003)	-	-
CompPerform←Financial Resources	0.185(0.001)	-	-
CompPerform←Technology Resources	0.029(0.538)	-	-
Product Devep← Size of firms	0.038(0.484)	-	-
Product Devep←Age of managers	0.014(0.823)	-	-
Product Devep←Educational Background	-0.060(0.324)	-	-
Product Devep←Financial Resources	0.169(0.027)	-	-
Product Devep←Technology Resources	-0.039(0.486)	-	-

NPD = New product development, the values in bracket show p values

### 4.6 Interviews results

Since cross sectional evidence are often criticized for being lack of validity and social desirabltiy biases. Moreover, respondents are restricted to provide answers of only limited options instead of allowing them to give their desire information. In order to reduce the bias and gain comprehensive information, we used a mixed method approach in this research. We conducted a face-to-face interview with 16 marketing professionals/managers (randomly from the survey responses) to articulate the implications and consequences in a useful way. We asked several questions, and their responses are discussed below.

1. Do you think your IC skill facilitates you in building new products that result in high profitability?
  Ans: There were 10 managers who answered “yes”, 3 managers said “partially” and 3 managers said “no”. Overall, majority of the managers were believed in their IC skills for building new products that can contribute to firm profitability.
2. Do you consider your financial skills such as financial terms such as interest rate, financial management and income statement in NPD?
  Ans: only 8 managers were agreed that their financial skills and literacy help them in building new products, 3 managers were partially agreed while 5 managers were disagreed. It indicates that financial skills of marketing managers are not a substantial factor for NPD.
3. Does your experience help you in NPD that results in high business performance?
  Ans: In response to experience, 9 managers said that their experience is worthy for NPD, 5 were partially agreed while only 2 were disagreed with the importance of experience in NPD. Overall, it displays that experience is important for NPD in the competitive edge.
4. What is your most vital skill in NPD out of IC, FL, and BE?
  Ans: In response to this question, the majority of the managers (11) were believed in their IC that is important for building new products; other managers were referred financial skills and experience.
5. What other skills do you think that are important for NPD, which results in high performance?
  Ans: We received mixed responses for this question. For instance, some managers said that marketing creativity and entrepreneurial skills are very useful for building new products. Some managers said that having customers information about their needs and wants can facilitate managers in building effective products. They also suggested that should be proper training and seminars on market information and market trend in order to know the customers’ needs and wants.
6. What kinds of policies, resources, and strategies do you think that can help in NPD, which results in high performance?
  Ans: We also got different suggestions and responses for this question. For instance, some marketing managers said that modern and advanced technology is essential for NPD. Other said that there should be adequate material and an effective infrastructure to build new products. Managers said that firms need to have strong research and development department to know the customers choices and to manage resources for building new products. Moreover, they also suggested that there should be active response on customers’ complaints in order to smooth the process of NPD.

## 5. Discussion and conclusion

This paper assessed the importance of intangible skills of marketing managers in NPD that in turn, facilitates SCP in SMEs sector. Though considerable number of studies have tested the importance of intangible skills and capabilities in SCP [106, 107] and innovative performance [119, 120]. However, in particular, the importance of the intangible skills; IC, FL and BE in SCP with mediating role of NPD has been missed. Moreover, the KBV and RBV theories have received minor attention in the marketing literature from emerging economies. Hence, this study contributes to existing literature of IC, FL, BE, NPD and SCP by employing the KBV and RBV theories. For instance, the KBV theory suggests that managers with sufficient knowledge, skills, and information benefits their firms in term of profitability and sustainable competitive advantage over other firms which have less competent and lack of skilled managers [17]. However, scholars from emerging markets have not tested this theory in terms of the marketing managers’ intangible skills. This study considers the IC, FL and BE as intangible assets and capabilities that are used by marketing managers for NPD that, in turn benefits SCP. Moreover, RBV theory unleashes the importance of tangible and intangible assets in sustainable competitive advantage and superior performance [17]. Recent studies have given more weight to the intangible resources (skills, knowledge, information, and capabilities) that can spur superior performance [16, 121]. Surprisingly, studies have ignored the intangible skills of marketing managers in building new products that, in turn enhance competitive performance in SMEs. Our study tested the skills such as IC, FL and BE to unbridle what kind of skill is important for high performance and NPD.

Our findings confirmed that the IC skill of marketing managers is a significant predictor of NPD and SCP. The findings favor other studies such as Agostini, Nosella, and Filippini [41] who revealed that managers use their IC to promote innovation and gain high profitability. Similarly, Ying, Hassan, and Ahmad [15] also revealed that SMEs managers emphasize their IC (being an intangible resource, less expensive and more convenient) due to limited resources and lack of support from external environment. However, studies have over-emphasized the relationship between IC and innovative performance [66, 119, 120] and have revealed a significant relationship. Our study matches such findings and argues that IC is also a crucial indicator of NPD in emerging economies.

Our results also confirmed that FL significantly contributes to NPD and SCP in SMEs sector. Our findings match several studies which have scrutinized a significant positive association between FL and sustainable competitive performance [51, 56] and innovative performance [122]. Consistent with Memon, Yong and Memon [123] who revealed that financial literature managers have high capabilities of exploiting new opportunities and generating new ideas that are very helpful for high performance.

Our study concluded that experienced managers have superior abilities in building new products and getting high performance. In line with Oura, Zilber, and Lopes [59]who showed that experienced managers know the market conditions, trends, customers’ needs, and in turn, these spur financial performance.

Our study scrutinized that NPD fully mediates the association between IC and SCP while it partially affects the path between FL, BE, and SCP. The findings strongly match Khan, Yang, and Waheed, [16], who stated that IC first builds sustainable competitive advantage that results in high performance. However, our results do not fully supported Ying, Hassan and Ahmad [15] because their results show a partial mediating role of resource acquisition between IC and sustainable competitive position in SMEs sector. To summarize, we argue that NPD is an important mediator between the intangible skills of the marketing managers and SCP.

### 5.1 Implications for practices

This research suggests several prominent implications specifically for marketing managers and also for professionals, CEOs (in hiring marketing staffs) and owners, and managers of business organizations. First, we emphasized how the intangible skills, IC, FL and BE are used as key elements for NPD. Findings from our research revealed that out of the three intangible skills, FL had the greatest influence on SCP, and IC had the greatest impact on NPD.

Hence, SMEs with scared resources can focus on these skills to build new products and to gain SCP.

Second, we scrutinized that all the three intangible skills of marketing managers are prominent for NPD and SCP in the SMEs sector. Therefore, SMEs should take action to increase the intangible skills of their marketing managers, which will help in NPD and result in gaining SCP. Because our study confirmed that NPD is a significant mediator, we argue that firms with skilled marketing staff can proactively seize NPD opportunities in the markets that result in SCP. For instance, within an integrated NPD department, marketing managers should communicate their ideas, knowledge, and information with their peers, so all the professionals will able to spur the process of NPD that is a piston for SCP. SMEs need to build an exchange mechanism in NPD departments to promote the regular communication, viewpoints, and ideas among the marketing managers and staff. Consequently, regular communication among all the departments (marketing, financial, production, sales and R&D, etc.) is also a pathway to NPD. For instance, as concluded in this study that FL and BE facilitate marketing managers in NPD. Hence, communizing knowledge (financial) and experience among various departments boosts the skills of marketing managers, which are worthy for NPD. Our study inspected that NPD fully mediates the relationship between IC and SCP while it plays a partial mediating role between FL, BE and SCP. Hence, considering the specific findings, we recommend SMEs to emphasize on IC skills of marketing managers if they aim for NPD. However, all these skills, IC, FL and BE should be focused if the main goals of the firms are NPD and SCP.

We have also controlled for tangible resources and demographic factors in the model that have provided mixed results. For instance, we revealed that financial resources are very important for SCP and NPD as compared to technological resources. Parsimoniously, our study advises that SMEs need to balance their financial resources for NPD and SCP. Additionally, we found that the educational background and age of managers are the significant predictors of SCP. The implications are not only restricted to SMEs but also listed firms, as well as large organizations, can equally be benefitted. Moreover, the findings can be pedestrian for SMEs, and listed firms work in European and developed markets.

### 5.2 Limitations and pathways for future studies

In spite of the fact that this study discussed several important implications for marketing managers, SMEs, and policymakers, there are still some constraints of this study. For instance, we collected data from marketing managers working in SMEs sector while ignored the marketing managers of the listed firms. It is advised to survey managers from listed and large firms to articulate better results. Similarly, we encourage researchers from other emerging markets to expand the model in their industrial sector to gain comprehensive insights. We have used only three intangible skills in this research. Indeed these three vary very effective and prominent in case of SMEs. However, other intangible skills such as creativity and leadership qualities can influence new product development, as pointed out by Cheng and Yang [124]. Hence, we recommend considering the leadership styles and creativity in the model to eloquent the discoveries. Similarly, entrepreneurial ability of marketing managers can be considered for future models to know which types of entrepreneurial skills are essential and useful for NPD. Another zone for future studies is testing demographic characteristics; age, gender, education, experience, etc. of managers that are controlled in this research. Testing these factors facilitate business organizations what kinds of managers should be retained in marketing department for building new products. Another recommendation for future studies is to test the distinguish role of tangible and intangible resources in NPD. However, we give more worth to this zone in listed and large firms as SMEs often believe in intangible skills that is the core theme of this research. It will enable marketing department of listed firms in developed and emerging economies to make strategies for new products.

To conclude the findings, our research revealed that all intangible capabilities, IC, FL, and BE of marketing managers are very crucial for NPD and SCP. Moreover, NPD fully mediates the path between IC and SCP while it partially affects the association between FL, BE and SCP. In the tangible resources, financial resources are significant, while technological resources are not very important in SMEs sector.

## Supporting information

S1 Appendix (DOCX)
