# Correction: Unpacking the importance of intangible skills in new product development and sustainable business performance; strategies for marketing managers

**DOI:** 10.1371/journal.pone.0257714

**Published:** 2021-09-23

**Authors:** Salman Ali, Guihua Li, Ping Yang, Kramat Hussain, Yousaf Latif

The affiliation for the fourth author is incorrect. Kramat Hussain is not affiliated with #2 but with #3: College of Management and Economics, Tianjin University, P.R. China.

## References

[1] Ali S, Li G, Yang P, Hussain K, Latif Y (2020) Unpacking the importance of intangible skills in new product development and sustainable business performance; strategies for marketing managers. PLoS ONE 15(9): e0238743. 10.1371/journal.pone.0238743 32976509 PMC7518595

