# Supplementary material for: Unpacking the importance of intangible skills in new product development and sustainable business performance; strategies for marketing managers
Source: PLoS One. 2020 Sep 25;15(9):e0238743. doi: 10.1371/journal.pone.0238743 (PMC7518595; doi:10.1371/journal.pone.0238743)
Supplement: S1 Appendix — (DOCX) [file pone.0238743.s001.docx]

**Appendix**

Intellectual capital: source [15, 42]

|  | Our organization has a clear view of our current core knowledge |
| --- | --- |
|  | Our organization has a clear view of what knowledge and competences are the most relevant for the objectives |
|  | Our organization’s knowledge and competences are evaluated systematically |
|  | Our organization benchmarks our strategic knowledge against that of our competitors |
|  | Our organization explicitly recognizes knowledge as a key element in the strategic planning exercises |
|  | Our organization has a clear strategy for developing knowledge and competences |

Financial literacy: source [15]

|  | We have the ability to analyze our financial performance periodically |
| --- | --- |
|  | My enterprise makes monthly income returns to the lender |
|  | I receive training on proper book keeping skills |
|  | My enterprise has bought formal insurance for our businesses |
|  | The management of this business can compute the cost of its loan funds |
|  | My enterprise operates a savings account |
|  | The entrepreneur can prepare basic books of accounts |
|  | The firm is aware of the operations of lending firms relating to our financial needs |
|  | Am aware of the costs and benefits of accessing credit |
|  | The firm is able to correctly calculate interest rates on my loan payments |
|  | We have required skills to ascertain the financial trends of the firm |
|  | We have skills of minimizing losses by minimizing bad debts |
|  | The manager of this business has basic accounting knowledge |

Business Experience: source [15]

| 1. Domestic business marketing experience |
| --- |
| 1. Exports and imports experience |
| 1. Problem-solving experience |
| 1. Decision-making experience |
| 1. Marketing resource management and utilization experience |

New Product Development: source [110]

| 1. An innovation…a totally new product to the world that opened up a brand new market |
| --- |
| 1. A totally new product to the world, but one where there was an existing market, i.e., replaced other products |
| 1. A totally new product to your company that offers new features to the market (existing market) |
| 1. A new product line to your company (existing market and existing products sold by others) |
| 1. A new item in an existing line. |
| 1. A significant modification of an existing product. |

Sustainable Competitive Performance: source [42, 111].

| 1. Return on investment (ROI) |
| --- |
| 1. Profits as a percentage of sales |
| 1. Decreasing product or service delivery cycle time |
| 1. Rapid response to market demand |
| 1. Rapid confirmation of customer orders |
| 1. Increasing customer satisfaction |
| 1. Increasing profit growth rates and growing market shares |
| 1. In reducing operating costs |
